# Next-Generation Sequencing Identifies the Danforth's Short Tail Mouse Mutation as a Retrotransposon Insertion Affecting *Ptf1a* Expression

**DOI:** 10.1371/journal.pgen.1003205

**Published:** 2013-02-21

**Authors:** Christopher N. Vlangos, Amanda N. Siuniak, Dan Robinson, Arul M. Chinnaiyan, Robert H. Lyons, James D. Cavalcoli, Catherine E. Keegan

**Affiliations:** 1 Department of Pediatrics, University of Michigan, Ann Arbor, Michigan, United States of America; 2 Center for Translational Pathology, University of Michigan, Ann Arbor, Michigan, United States of America; 3 Department of Pathology, University of Michigan, Ann Arbor, Michigan, United States of America; 4 Biological Chemistry Department, University of Michigan, Ann Arbor, Michigan, United States of America; 5 University of Michigan DNA Sequencing Core, University of Michigan, Ann Arbor, Michigan, United States of America; 6 Center for Computational Medicine and Bioinformatics, University of Michigan, Ann Arbor, Michigan, United States of America; 7 Department of Human Genetics, University of Michigan, Ann Arbor, Michigan, United States of America; The Jackson Laboratory, United States of America

## Abstract

The semidominant Danforth's short tail (*Sd*) mutation arose spontaneously in the 1920s. The homozygous *Sd* phenotype includes severe malformations of the axial skeleton with an absent tail, kidney agenesis, anal atresia, and persistent cloaca. The *Sd* mutant phenotype mirrors features seen in human caudal malformation syndromes including urorectal septum malformation, caudal regression, VACTERL association, and persistent cloaca. The *Sd* mutation was previously mapped to a 0.9 cM region on mouse chromosome 2qA3. We performed Sanger sequencing of exons and intron/exon boundaries mapping to the *Sd* critical region and did not identify any mutations. We then performed DNA enrichment/capture followed by next-generation sequencing (NGS) of the critical genomic region. Standard bioinformatic analysis of paired-end sequence data did not reveal any causative mutations. Interrogation of reads that had been discarded because only a single end mapped correctly to the *Sd* locus identified an early transposon (ETn) retroviral insertion at the *Sd* locus, located 12.5 kb upstream of the *Ptf1a* gene. We show that *Ptf1a* expression is significantly upregulated in *Sd* mutant embryos at E9.5. The identification of the *Sd* mutation will lead to improved understanding of the developmental pathways that are misregulated in human caudal malformation syndromes.

## Author Summary

Birth defects are the leading cause of infant mortality in the United States, accounting for 1 in 5 infant deaths annually. Birth defects that affect development of the caudal portion of the embryo can include malformations of the spine, such as spina bifida, and malformations of the kidneys and lower gastrointestinal tract. Little is known regarding the genetic causes of human caudal birth defects. The Danforth's short tail (*Sd*) mouse shares many similarities with these caudal birth defects that occur in humans. In this manuscript, we used next-generation sequencing to identify the genetic cause of the *Sd* mouse phenotype. We found that the *Sd* mutation is a retrotransposon insertion that inappropriately turns on a nearby gene that is normally important for pancreas development. Future studies of *Sd* mice will help us understand the pathogenesis of caudal birth defects in humans.

## Introduction

The Danforth's short tail (*Sd*) mouse mutation arose spontaneously in the early 1920s in an inbred mouse colony maintained in the laboratory of C.H. Danforth [1], [2]. The inbred line in which the *Sd* mutation arose was being maintained for study of a dominant but incompletely penetrant posterior duplication phenotype. Danforth shared four mice with shortened tails (2 males and 2 females) with L.C. Dunn and S. Gluecksohn-Schoenheimer. None of the offspring of these shared mice displayed the posterior duplication phenotype, indicating that Danforth's original line was segregating two different mutations [1], [2]. The new short tailed line was named short-Danforth or *Sd*. Dunn *et al.* determined that the *Sd* mutation was inherited in a semi-dominant manner with complete penetrance and was not allelic to Brachyury (*T*) [2].

The *Sd* mutation causes severe defects in development of the axial skeleton, urogenital, and gastrointestinal systems [3]. Homozygous mutant mice (*Sd^sd/sd^*, to be denoted herein as *Sd/Sd*) are born live with viability equal to their littermates, although death occurs within 24 hours of birth [2], [3], [4]. The phenotype of homozygous mutants includes complete lack of tail development with a shortened spinal column due to absence of caudal and sacral vertebrae. The kidneys are completely absent, though occasionally a single small kidney rudiment can be identified medially. The bladder is present, but there is no urethral opening. The cloaca persists due to lack of proper growth of the urorectal septum to divide the cloaca into the primary urogenital sinus (ventrally) and the rectum (dorsally). This septation defect also results in a blind ending intestine and anal atresia.

Heterozygous animals *(Sd^sd/+^*, denoted herein as *Sd/+*) are less severely affected than homozygotes, and survive into adulthood [2], [3]. Adult heterozygotes are fertile, but fecundity is reduced. The heterozygous phenotype is 100% penetrant and includes caudal and sacral skeletal malformations and the characteristic extremely shortened tail. Both urogenital and anal openings are present. The kidneys are variably affected and are generally smaller in size (unilaterally or bilaterally), and unilateral kidney agenesis is not uncommon [4]. However, histologically the kidney tissue is normal. Despite this knowledge, it has been hypothesized that the kidney defects are the cause of reduced lifespan in *Sd*/+ mice [2].

During embryogenesis *Sd/Sd* mutants develop normally through approximately embryonic day (E)10.0–10.5, when the first external manifestation of the phenotype is shortening of the tail [4], [5], [6]. Histologically the phenotype first observed in *Sd/Sd* embryos at E9.5 is disintegration of the notochord [4], [6], [7], and no new notochord is formed caudally from this point [6], [7]. Additionally, the chordal cells remain abnormally close to the neural tube. By E14 the notochord has completely disintegrated except for a few fragments remaining in the sacral region. However, any floor plate that was properly formed remains. The notochord degeneration is equally severe in both homozygous mutant and heterozygous embryos, but it starts slightly earlier in *Sd/Sd* animals (E9.5) than in *Sd*/*+* embryos (E10.5) [6], [7].

The phenotype of *Sd* mice resembles several human caudal developmental disorders characterized by malformations of the spine, lower gastrointestinal, and urogenital systems. These include caudal regression syndrome (CRS, OMIM #600145), urorectal septal malformation sequence (URSMS, also known as persistent cloaca), Currarino syndrome (OMIM #176450), and VACTERL (Vertebral-Anal-Cardiac-Tracheo-Esophageal fistula-Renal-Limb anomalies) association (OMIM #192350). Currarino syndrome, characterized by the triad of partial sacral agenesis, presacral mass, and anorectal malformation, is caused by mutations in the *MNX1* (*HLXB9*) gene in 50% of sporadic and 90% of familial cases with the classic phenotype [8], [9], [10]. In addition, private mutations in *VANGL1* [11], *ZIC3* [12], *HOXD13* [13], and *PTEN* [14] have been described in single cases of caudal dysgenesis and/or VACTERL phenotypes. However, the developmental mechanisms that lead to caudal malformations in humans are still largely unknown. Because of the significant overlap in phenotype, *Sd* mice are an ideal model to improve our understanding of the genetic and pathophysiologic mechanisms that lead to human caudal malformations.

Although the *Sd* mutation arose over 90 years ago and the mutation has been genetically mapped to a small region on mouse chromosome 2 [15], the specific genetic lesion has not yet been identified. Here, we report the identification of the Danforth's short tail mutation. We used the flanking markers from the previously published genetic map to delineate the corresponding physical map of the *Sd* critical region. Direct DNA sequencing of all the exons and exon/intron boundaries of the positional candidate genes and expressed sequence tags (ESTs) did not reveal any mutations. Since direct sequencing of the exonic DNA only provided ∼1% coverage of the *Sd* critical region, we performed next-generation sequencing (NGS) of the entire *Sd* genomic interval. Standard bioinformatic analysis of our NGS data did not reveal any causative mutations; therefore, we interrogated reads for which only a single end of a paired-end sequence mapped correctly to the *Sd* locus. Using this innovative technique, we identified an early transposon (ETn) retroviral-like insertion at the *Sd* locus. Expression analysis at E9.5 revealed that the *Sd* ETn causes inappropriate expression of the *Ptf1a* gene at this developmental timepoint.

## Results

### The *Sd* critical region and candidate gene analysis

**Figure 1:**
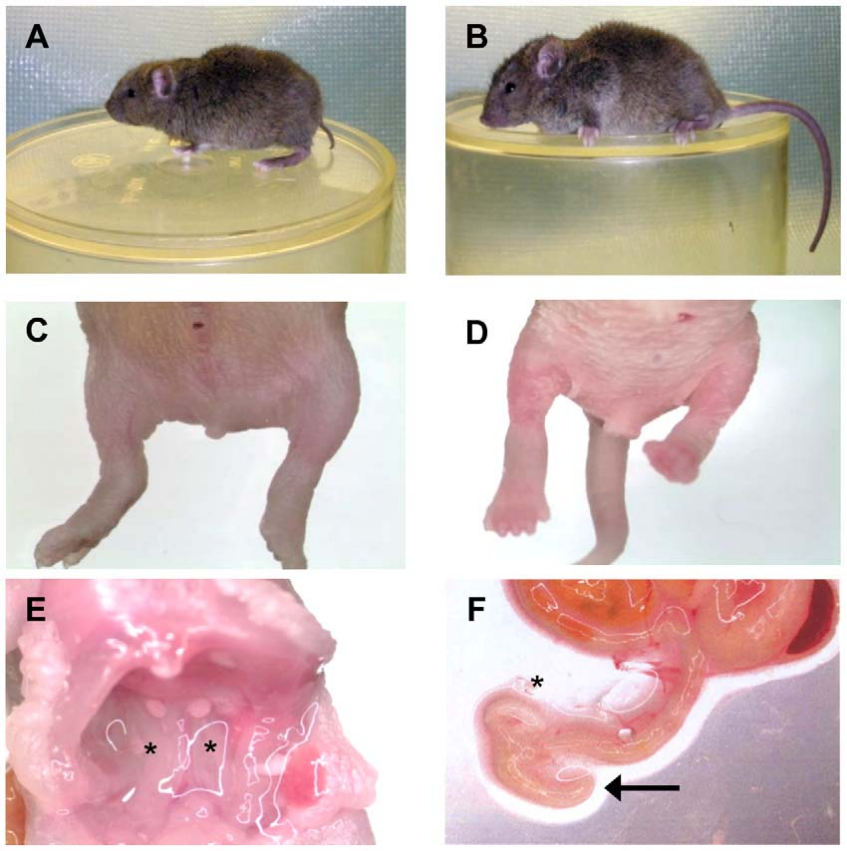
The *Sd* phenotype. A) Heterozygous (*Sd*/+) mouse. B) Wildtype (+/+) mouse. C) Homozygous (*Sd/Sd*) P0 neonate. Note the lack of urogenital openings and tail. D) Wildtype (+/+) P0 neonate. E) Homozygous (*Sd/Sd*) mutant neonate showing bilateral kidney agenesis. Kidney location indicated by asterisks below the normally formed adrenal glands. F) Homozygous (*Sd/Sd*) mutant intestine. The colon ends blindly (indicated by arrow), never connecting to the rectum which is never formed (see panel C). The asterisk indicates normal formation of the caecum.

**Figure 2:**
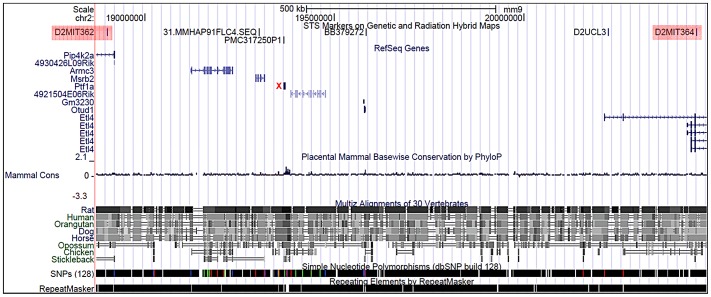
Physical map of the *Sd* critical region as viewed on the UCSC genome browser ( http://www.genome.ucsc.edu **).** The *Sd* region on chromosome 2 is defined by the flanking genetic markers D2Mit362 proximally and D2Mit364 distally (markers are highlighted in red) and represented by bases 18,686,882–20,915,358 on the July 2007 genome build (mm9). The *Sd* critical region contains 11 known genes. The mutation location is denoted by the red X.

We examined the *Sd* mutation on the recombinant inbred RSV/LeJ mouse line, and have confirmed the previously published phenotype of *Sd/+* and *Sd/Sd* mice (Figure 1). The Danforth's short tail mouse phenotype is characterized by significant anomalies of the urogenital, digestive, and skeletal systems, and is 100% penetrant. Using backcrossing methods, Alfred *et al.* previously mapped the *Sd* critical region to an ∼0.9 cM region on mouse chromosome 2qA3 [15]. Two groups of flanking markers defined the proximal and distal borders; however, the mapping was not able to distinguish between the individual markers in each group. We used the flanking marker groups from the published genetic map to identify the corresponding *Sd* critical region on the mouse physical map. Using the UCSC Genome browser (July 2007; NCBI37/mm9) we defined the *Sd* critical region as 1.5 megabase pairs (Mb) spanning Chr2:18,901,614–20,456,182 flanked by the genetic markers D2Mit362 and D2Mit364 (Figure 2). The critical region contains 9 annotated RefSeq genes, representing both coding and non-coding genes. In addition, we identified 3 candidate expressed sequence tags (ESTs) not represented by RefSeq genes in the critical region (Figure 2; Table 1).

**Table 1:** Genes and ESTs mapping to the *Sd* critical region.

Gene ID	GenBank Acc.	Gene name and Function	# Exons	References
*Pip4k2a*	NM_008845	Phosphatidylinositol phosphate kinase 2a; associated with schizophrenia and hemoglobin H disease in humans	10	[50], [51]
4930426L09Rik	NR_024323	Novel EST, No open reading frame	1	
*Armc3*	NM_001081083	Novel, predicted to be involved in Wnt signaling by presence of alpha-helical armadillo repeats	20	
AK076779	AK076779	Novel EST, No open reading frame	4	
*Msrb2*	NM_029619	Methionine sulfoxide reductase 2; repairs methionine residues in proteins that have been oxidatively damaged. The *Msrb* genes are predicted to play a protective role against amyloid diseases such as Parkinson and Alzheimer diseases.	5	[52], [53], [54]
BQ085140	BQ085140	Novel EST, contains no open reading frame	2	
AK053418	AK053418	Novel EST, No open reading frame	4	
*Ptf1a*	NM_018809	Pancreas specific transcription factor 1a; known to be necessary for proper development of the pancreas and cerebellum in mice and humans	2	[25], [26], [27], [28]
*4921504E06Rik*	NM_027600	Novel, Contains open reading frame, no protein domains identified	17	
Gm3230	NR_033642	Novel, contains no open reading frame	1	
*Otud1*	NM_027715	Novel, predicted to be a deubiquitinating enzyme, contains OTU (ovarian tumor)domains)	1	
*Etl4*	NM_001081006.1	Enhancer trap locus 4, no function identified, gene trapped alleles have skeletal anomalies	20	[55], [56]

Using template *Sd/Sd* DNA from the RSV/LeJ line we individually sequenced a total of 87 annotated exons and intron/exon boundaries mapping to the *Sd* region. We compared sequences from the PCR products to the C57BL/6J (B6) mouse reference genome sequence and identified only 6 DNA changes. Among these changes, we identified a novel microsatellite in intron 9 of the *4921504E06Rik* gene (IVS9+76_77DupTATC). Homozygous *Sd* mice have 16 copies of a TATC repeat, while *Sd*/+ mice have one allele with 16 copies and one with 15 copies, and wildtype mice from the RSV/LeJ line and the B6 reference genome have 15 copies. This microsatellite segregated with the mutant phenotype in all mice/embryos tested (26/26). Although linked to the *Sd* mutation and serving as a genetic marker for the *Sd* mutation, the microsatellite itself is unlikely to be mutagenic since it does not change any coding sequence or uncover a cryptic splice site. In addition, we tested 11 mouse lines and found that the A/J, CBA, CD-1, and DBA mouse lines also carry 16 copies of the TATC repeat, and the CAST/EiJ line has 18 copies, indicating that this TATC repeat is a polymorphic microsatellite (data not shown).

Our exon sequence analysis of homozygous mutant *Sd* DNA from the RSV/LeJ recombinant inbred line also identified 5 SNPs in exon 2 of the *Etl4* gene. Three of these changes were represented in the SNP database (dbSNP: rs33065171, rs33061041, rs32925809), and two changes were novel (c.207A>G and c.210G>A). These 5 SNPs were homozygous in all mice from the RSV/LeJ recombinant inbred line regardless of phenotype (*Sd/Sd*, *Sd/+*, and *+/+*). Thus, no mutagenic DNA changes were identified in direct sequencing of the annotated exons mapping to the *Sd* critical region.

### DNA capture/enrichment of the *Sd* critical region and next-generation sequencing

Since PCR and direct DNA sequencing of all known exons mapping to the critical region failed to identify the *Sd* mutation, we employed next-generation sequencing (NGS) technology for mutation detection. DNA capture/enrichment allowed us to focus only on the *Sd* genomic interval on mouse chromosome 2. An oligonucleotide based array was designed to capture the DNA mapping between bases 18,883,257 and 20,481,194 on mouse chromosome 2. Repeat masking software was employed to ensure only unique DNA sequences would be present on the array. Total genomic DNA isolated from a phenotypically heterozygous (*Sd*/+) neonatal mouse was hybridized to the capture array.

Prior to performing the NGS reaction we confirmed success of the DNA capture by quantitative real time PCR (qRT-PCR) experiments (Figure S1). Using two sets of primer pairs, one mapping within the captured region (to *Etl4*, exon 2), and the other mapping outside the captured region (to mouse β-Actin) we demonstrated an ∼20 fold increase in captured DNA, indicating a successful enrichment of the *Sd* interval genomic DNA sequences.

We performed 36 base pair paired-end sequencing using one lane on an Illumina Genome Analyzer IIx. The sequencing reaction produced 1.8 Gb of DNA sequence. Assembly and alignment of our NGS data was performed using the Bowtie software program [16]. We used the B6 genome as a template for the alignment and analysis of the NGS data. 93% of the obtained sequence reads mapped to the captured region on mouse chromosome 2 with both paired-ends mapping to the proper position and in the proper orientation. The sequence reads obtained cover 811,821 bases (51%) of the 1.5 Mb *Sd* critical interval as defined by Alfred *et al.* [15], and the average coverage of sequence reads across the interval was 443×. The *Sd* critical region is highly repetitive, and ∼49% of the region is made up of LINE, SINE and other highly conserved repetitive DNA sequences. In order for optimal capture and sequence alignment these DNA sequences were excluded from our capture array and are not fully represented in our data. Since we used heterozygous DNA as a template for sequencing, we set an initial variant threshold for our mutation analysis to be 40% of reads (meaning any change from reference showing up at least 40% of the time would trigger a heterozygous call). In the initial data analysis we did not identify any point mutations or small insertions or deletions. We increased the heterozygous call ratio to 25%/75% (where if 25% of reads differed from the reference a heterozygous call would be denoted) and still did not identify any mutations.

We hypothesized that the *Sd* mutation might be due to insertion of a retrotransposon. Retrotransposon insertions are frequently mutagenic in mice and are estimated to be the cause of 10% of spontaneous mouse mutations [17], [18]. In order to search for novel retrotransposon insertions, we sought to align separately individual ends of each pair from our NGS data. Reads for which one of the paired-ends mapped to the *Sd* critical region and the other end failed to align to unique (non-repeat masked) DNA were filtered to remove failed reads and low complexity sequences and screened for repetitive elements. Although our capture array was designed to exclude repetitive DNA, we expected some carry over, especially of paired-end reads where one end of the fragment mapped to unique DNA. New Perl scripts were written (available from the authors) to accomplish the analysis. Using this method we discovered a region in which one end of 166 independent paired-end reads mapped in the proper orientation to a consistent chromosome 2 position, and the other end of the pair mapped to the 5′ end of an ETn specific retrotransposon long terminal repeat (LTR) sequence. In addition, since the DNA sample sequenced came from a heterozygous mouse, we were also able to identify 1127 reads from the wildtype allele at the same location showing no variation from the reference genome. There are a larger proportion of reads representing the wildtype allele likely due to reduced capture efficiency from the mutant allele since repetitive sequences were excluded from the capture array. The method presented here provides a way to utilize “unmapped” reads which would normally be discarded. These unmapped data are important to understand the complexity of the genome, and the variation in individuals or strains from a reference sequence.

### The *Sd* mutation is an ETn insertion

**Figure 3:**
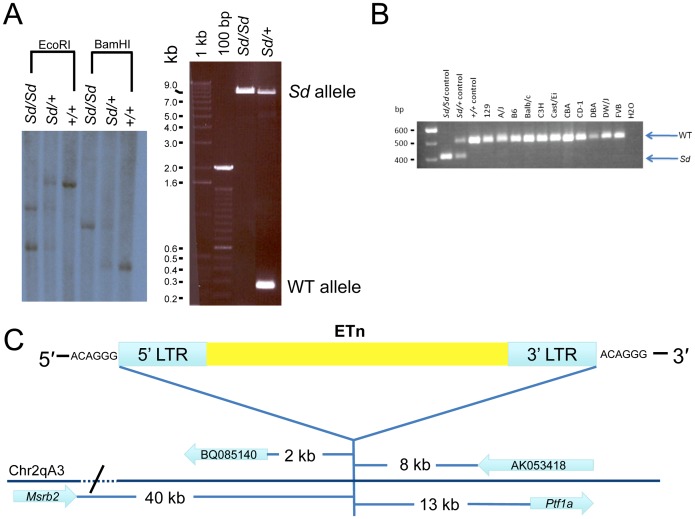
Confirmation and mapping of the *Sd* mutation. A) Southern analysis and PCR showing the presence of a large DNA insertion at the *Sd* locus. B) Multiplex PCR from inbred mouse lines showing the mutation is only present in *Sd* mice, and is not a polymorphism. In this three primer PCR reaction the *Sd* amplimer is 406 bp, while the WT amplimer is 510 bp. C) Mapping of the *Sd* mutation which we identified as an ETn (early transposon) in relation to nearby gene/ESTs, figure not to scale.

To confirm the presence of an ETn insertion at the *Sd* locus we performed both Southern analysis and long range PCR (Figure 3A). EcoRI and BamHI digested DNA isolated from *Sd/Sd*, *Sd/+*, and +/+ mice was hybridized with an ∼1000 bp probe from non-repetitive DNA flanking the location of the ETn insertion. The probe hybridized to a 7,300 bp fragment in wildtype DNA digested with EcoRI, which was predicted from the mouse genome reference sequence. However, hybridization of the probe to EcoRI digested *Sd/Sd* DNA resulted in detection of two bands of ∼4,000 bp and ∼6,000 bp, and hybridization to heterozygous DNA showed all three bands (Figure 3A). Similarly in BamHI digested DNA from a +/+ mouse, an expected band of 3,500 bp was detected. In an *Sd*/*Sd* mutant a single fragment of ∼5,000 bp was identified, and both BamHI fragments were detected in DNA from the *Sd*/+ mouse (Figure 3A).

To further confirm the presence of an ETn insertion at the *Sd* locus, long range PCR was utilized with PCR primers designed flanking the insertion site. Using wildtype genomic DNA as a template, the primers amplified a product of 253 bp as expected (Figure 3A). When *Sd*/*Sd* DNA was used as a template, the 253 bp product was absent, and instead a product of ∼9,000 bp was amplified. In heterozygous DNA, both fragments were amplified (Figure 3A). These data, in conjunction with the Southern analysis, confirmed insertion of a large piece of DNA at the *Sd* locus. The long range PCR product was cloned and sequenced using primer walking. The *Sd* insertion was identified as an early transposon (ETn) at mouse chromosome 2 position 19,355,026. There was no loss of wildtype DNA at the insertion site. However, the insertion resulted in a 6 base pair terminal duplication sequence (TSD) flanking the ETn. TSD sequences are a hallmark of retroviral insertion. Sequencing data showed that the long terminal repeats (LTRs) of the ETn were 847 base pairs in length and 100% identical to each other. The internal sequence of the ETn is 6,834 bp in length and does not contain any open reading frames. The total length of the *Sd* ETn is 8,528 bp (GenBank Accession JX863104).

Rigorous comparison of our next-generation sequencing data across the *Sd* critical region to the reference B6 genome, and available sequence from the CBA genome (CBA is the last known outcross of the RSV/LeJ line), did not reveal any significant sequence changes. Thus, we were not able to identify the mouse strain on which the *Sd* mutation originally arose in the 1920s. In order to rule out the possibility that the *Sd* ETn is a common polymorphism, we assembled a panel of DNA from 10 inbred and 1 outbred mouse strains for analysis. These strains were chosen to include a diverse number of strains spanning many arms of the inbred mouse genealogy chart [19]. We specifically included the A/J line since ETn sequences are reported to be highly active in this strain [17]. We designed a locus specific multiplex PCR and screened the 11 additional mouse strains (Figure 3B). The *Sd* ETn was not identified in any of the strains, indicating that it is not a common strain polymorphism.

### q–RT PCR and 5′ RACE analysis

**Figure 4:**
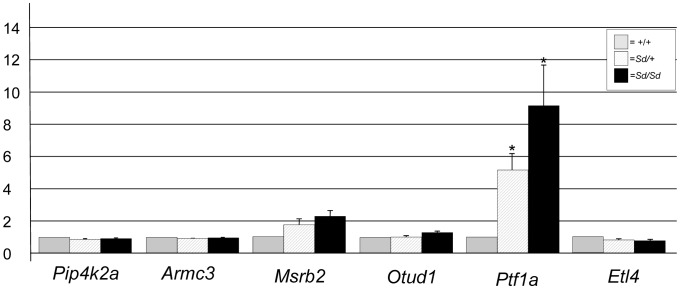
Gene expression at E9.5 in *Sd* mutant embryos. Quantitative real time PCR plot of gene expression in RNA isolated from *Sd/Sd* (black bars), *Sd/*+ (hatched bars), and +/+ (grey bars) embryos of RefSeq genes mapping to the *Sd* interval. Y-axis shows fold change normalized to 1 for wildtype (+/+) expression. * denotes significance in expression levels where p<0.05 (For *Ptf1a*; *Sd*/+ versus +/+ p = 0.029, *Sd/Sd* versus +/+ p = 0.044).

ETn sequences are known to affect gene expression when they insert within a gene and are predicted to affect expression of genes when they land upstream [17]. The *Sd* ETn insertion is located 12,463 bases upstream of the *Ptf1a* start codon and within the gene's previously defined 15.6 kb promoter/enhancer region and in the same orientation as the *Ptf1a* gene (Figure 3C; Figure S2) [20]. Thus, we hypothesized that *Ptf1a* expression is affected by the ETn insertion. qRT-PCR on RNA isolated from whole embryos at E9.5, when the first manifestation of the *Sd* phenotype becomes apparent, indicated the *Ptf1a* gene is upregulated at ∼9 times normal levels in *Sd*/*Sd* embryos at this timepoint (Figure 4). This over expression was also seen in *Sd/+* embryos where the *Ptf1a* is expressed ∼5 fold that of wildtype (Figure 4). These data demonstrate a dose-dependent up regulation of *Ptf1a* expression that correlates with the number of mutant alleles and the severity of the *Sd* phenotype.

RNA expression levels of other genes mapping to the *Sd* critical interval, including *Pip4k2a*, *Armc3*, *Otud1*, and *Etl4* were equivalent in *Sd/Sd*, *Sd/+*, and +/+ embryos (Figure 4). The *Msrb2* gene showed an ∼2 fold change that was not statistically significant (Figure 4). This increased expression change in *Msrb2* is likely due to our small sample size (n = 3/genotype). We were unable to amplify message from the *4921504E06Rik* gene at this timepoint. In addition, we identified an EST (BQ085140) that is not represented in the RefSeq data located 2 kb from the 5′ end of the ETn. BQ085140 is the closest expressed sequence to the ETn, however, its orientation is opposite that of the retrotransposon. We were unable to amplify BQ085140 from RNA isolated from E9.5 embryos of any genotype. EST AK053418 maps between the ETn insertion site and the *Ptf1a* gene, 8 kb from the 3′ end of the ETn and also in the opposite orientation. We were unable to amplify AK053418 from RNA isolated at this timepoint in either mutant or wildtype RNA. These data indicate that the two closest expressed sequences are not expressed at E9.5, and are not affected by insertion of the ETn at this timepoint even though *Ptf1a* is overexpressed. We did not test the expression of AK076779, 4930436L09Rik, and Gm3230 based on their distance from the ETn and lack of open reading frames.

To determine whether the ETn was acting as a promoter/enhancer or resulting in a dominant negative fusion RNA we performed 5′ RACE (rapid amplification of cDNA ends). Using RNA isolated from E9.5 *Sd*/*Sd* embryos, our 5′ RACE reactions only amplified the 5′ end of the *Ptf1a* gene (represented by GenBank Accession 007922). Thus, there is no incorporation of ETn sequence in the RACE products from homozygous mutant RNA. These data indicate that the *Sd* ETn is acting as an enhancer to strongly augment expression of normal *Ptf1a* transcripts.

### *Ptf1a* transgenic mouse lines

We generated two transgene constructs in an attempt to recapitulate the *Sd* phenotype (Text S1). In the first experiment the *Ptf1a* coding sequence was cloned into the pCAGGS vector [21]. The pCAGGS vector contains the cytomegalovirus immediate early enhancer and the chicken β-actin promoter, resulting in ubiquitous expression of cloned sequences in mammalian cells [21], [22]. Among 116 harvested embryos only 6 carried the pCAGGS-*mPtf1a* transgene, compared with a typical yield of 10 to 20% transgenic offspring [23] (Table S1). Three of the six transgenic embryos did not express *Ptf1a* ectopically and were phenotypically normal at E16.5. The remaining three transgenic embryos had arrested growth and were dead and in the process of resorbing (Table S1). These data are consistent with a negative effect of *Ptf1a* overexpression on development.

In the second experiment we used recombineering [24] to create a BAC-based genomic clone containing both the *Ptf1a* gene and the *Sd* ETn (Text S1). Using gap repair we sub-cloned a 31 kb DNA fragment containing the *Ptf1a* gene and the previously reported known promoter, 5′ enhancer, and 3′ control region sequences (Figure S2) [20]. We inserted the ETn into the precise *Sd* location 12 kb upstream of *Ptf1a* to create an ETn/*Ptf1a*-containing genomic transgene. Both the ETn-containing transgene and the control BAC-based transgene lacking the ETn were injected into fertilized mouse eggs. Founder (G0) embryos were dissected between E14.5 and E15.5. No abnormal phenotype was apparent in the transgenic embryos with or without the *Sd* ETn insertion. However, there were significantly fewer transgenic embryos (3 of 23; p = 0.0086, Fishers exact test) carrying the ETn-containing transgene than the associated control transgene (16 of 32) (Table S1).

## Discussion

Our data reveal that the *Sd* phenotype is caused by an insertion of an early transposon (ETn) at the *Sd* locus. The *Sd* ETn maps upstream of the promoter of the *Ptf1a* gene causing overexpression of normal *Ptf1a* transcripts at E9.5. No fusion transcript of the ETn and the *Ptf1a* gene was detected, indicating that the ETn is functioning as a strong enhancer. Ptf1a (originally termed p48) is a basic-helix-loop helix transcription factor that was first cloned from a rat exocrine-specific pancreatic cell line [25]. Northern blot analysis in adult rat tissues revealed expression limited to the exocrine pancreas [25]. Thus, p48 was given the name “pancreas specific transcription factor 1a.” Northern and PCR-based analysis in developing mouse pancreas initially showed onset of *Ptf1a* expression at embryonic day 12 (E12) [25]. However, further examination of whole mouse embryo by RT-PCR indicated that *Ptf1a* expression is highest at E10.5 [26]. Analysis of the expression pattern of *Ptf1a* using whole mount *in situ* hybridization indicated that expression of the gene is not restricted to the developing pancreas. High levels of expression were found at E10.5 in the cerebellar primordium and throughout the length of the developing neural tube in addition to the developing pancreatic buds [26]. In humans, recessive mutations of *PTF1A* cause cerebellar agenesis and permanent neonatal diabetes due to pancreatic agenesis [27].

*Ptf1a* null mice are born in Mendelian ratios; however, they lack a pancreas and die shortly after birth [28]. The exocrine cells of the developing pancreas fail to form in null embryos, and exocrine specific genes (amylase, etc) are never expressed. However, some endocrine cells do form in null embryos and express endocrine specific genes (insulin, etc.); interestingly, these endocrine cells reside in the spleen. Based on the null phenotype it was thought that *Ptf1a* was essential for development of exocrine-specific pancreas cells and that the exocrine cells were secondarily crucial for the proper spatial organization of the endocrine pancreas [28]. Fate mapping using a *Ptf1a*-Cre locus specific knock-in and cre-mediated LacZ expressing mice showed that *Ptf1a* expression was present in all early pancreatic precursor cells as nearly all acinar, ductal, and islet cells showed a history of *Ptf1a* expression [29]. *Sd* mice do not have a reported pancreatic phenotype. Our analysis of *Ptf1a* overexpression in *Sd* mice only focused on total RNA isolated from E9.5 embryos, which is ∼1 day prior to pancreatic bud formation. It is unknown whether the ETn insertion influences *Ptf1a* expression occurring in the pancreatic primordia, or older pancreas.

*Sd* mice exhibit neuronal patterning abnormalities, and there is a significant decrease in the number of motor neurons in the most caudal portions of the developing embryo [30]. This could be secondary to failure of development of multiple caudal tissues in *Sd* mice, or could result from up regulation of *Ptf1a*. Mouse *Ptf1a* mRNA was injected into one blastomere of two cell stage *Xenopus* embryos [26]. Presence of the mouse *Ptf1a* mRNA in the cells of the developing *Xenopus* embryos suppressed growth in the cells which become interneurons and primary sensory neurons [26]. Injected embryos were not analyzed beyond neural development at early stages, so it is unknown whether overexpression of mouse *Ptf1a* in *Xenopus* would have any caudal phenotypic characteristics similar to *Sd* mice. Overexpression of *Ptf1a* has not yet been studied in other model organisms. Thus, the *Sd* mouse is the first report of a phenotype caused by ectopic *Ptf1a* expression.

*Ptf1a* is a transcription factor required in several tissue lineages and which interacts with tissue specific co-factors [31]. *Ptf1a* is part of a heterotrimeric complex called PTF1 [25], [26], [32]. The PTF1 complex includes *Ptf1a*, RPB-J or RPB-L, and a class A bHLH protein (HEB, E2, or E47) [31]. The RPB genes act as both repressors and co-activators [33]. RPB-J is ubiquitously expressed and responsive to the Notch intracellular domain, while RPB-L is tissue-specific in the developing pancreas, neural tube, and cerebellum, and is Notch-independent [33]. Ectopically increased *Ptf1a* could act to affect downstream gene expression through heterodimeric binding to RBP-J and/or heterotrimeric binding with RPB-J and a class A bHLH protein, as both heterotrimeric and heterodimeric complexes have the ability to bind DNA [31]. It is also possible that excessive *Ptf1a* may lead to de-repression of RPB-J regulated genes. However, it is unclear whether this would occur without specific signals from the Notch intracellular domain. It will be interesting to determine whether ectopic overexpression of *Ptf1a* causes misexpression of downstream genes, possibly through interaction with RBP-J.

Endogenous retrovirus (ERV) sequences containing long terminal repeats (LTRs) make up ∼8–10% of the human and mouse genomes [17]. ERV elements transpose in a copy and paste method via transcribed RNA intermediates. In humans, LTR mediated ERV retrotransposition has not been described and these elements have never been implicated in human disease. However, LTR mediated ERVs are mobile in mice and account for ∼10–15% of all spontaneous mouse mutations [17]. Early transposons (ETn) are a subfamily of the ERVs which also include intracisternal A particles (IAP) and MusD elements [34]. ETn elements range in size from 2 kb to 8 kb, do not contain open reading frames, and are non-autonomous [34], [35]. Rather, retrotransposition of the ETn elements require proteins encoded by the related MusD ERV [34], [36].

The ETn elements are known to be very highly expressed between E3.5 and E7.5 in all cells [37]. This high level of ETn expression likely accounts for the high rate of transposition of these elements in mice. After E7.5, global ETn transcription decreases to less than 5% of that seen in the undifferentiated cells of the early embryo. The expression pattern post-implantation (between E7.5–13.5) becomes more limited to mostly non-differentiated cells [38]. However, it is interesting to note that at E9.5 there is intense expression of ETn sequences in the caudal portion of the neural tube and caudal somites. [38]. It is tempting to hypothesize that this caudally localized ETn driven overexpression of *Ptf1a* at E9.5 results in the severe caudal defects observed in *Sd* mutant mice.

Luciferase assays have been used in cultured cells to study the transcriptional activity of LTR sequences contained in ETn elements [39]. Promoter activity can be up to 200-fold higher from the same LTR cloned into undifferentiated compared to differentiated cells [39]. Mutagenic ETn elements published to date have all landed intronically within the genes they affect [17], [18]. These ETn elements cause aberrant splicing of the gene in which the ETn inserted, resulting in premature polyadenylation and protein truncation. The related IAP elements are known to have transposed upstream of genes and act as strong promoters, as in the agouti locus [40]. Similarly, the Dactylaplasia (*Dac*) mouse is characterized by an incompletely penetrant limb phenotype consisting of missing central digital rays. The *Dac* mutation has been identified as a MusD element that maps downstream of the *Fgf8* gene, and *Fgf8* expression is downregulated during limb development in homozygous mutants [41]. The *Sd* ETn landed within the well-studied promoter/enhancer region of the *Ptf1a* gene [20]. It is plausible that insertion of the ETn within *Ptf1a* could usurp endogenous promoter activity until the ETn is methylated, thus suppressing endogenous *Ptf1a* promoter activity at early times. Our data do suggest that the *Sd* ETn is acting as a strong enhancer by causing a dose-dependent increase in *Ptf1a* expression that correlates with the severity of the *Sd* phenotype.

We attempted to recapitulate the *Sd* phenotype using two different transgene constructs; one with the ubiquitously expressed strong pCAGGS promoter driving the *Ptf1a* cDNA, and one BAC-based transgene with insertion of the ETn element upstream of the *Ptf1a* gene. Neither of these transgenes recapitulated the *Sd* phenotype. We hypothesize that the pCAGGS-driven expression of *Ptf1a* was more deleterious than the *Sd* mutation because of more widespread, and possibly higher level, of *Ptf1a* expression. The BAC-based ETn-containing transgene could also be expressed in novel spatial and temporal patterns that do not recapitulate the *Sd* mutation. Our ETn-containing BAC-based transgene may also be lethal since there were significantly fewer embryos carrying the ETn-containing transgene than the control. Although we designed our BAC-based genomic transgene to contain the known upsteam and downstream regulatory elements of the *Ptf1a* gene [20], it is possible that additional sequences necessary for recapitulation of the *Sd* phenotype are required and were unknowingly excluded in the design of our experiment (Figure S2). Passage through the germline might also be required for proper methylation of the ETn element to mirror the mutagenic effects of the *Sd* ETn.

Development of the notochord is severely affected in *Sd* mutant embryos [4], [7]. The notochord and associated floor plate develop normally through E9.5 in *Sd/Sd* embroys and E10.5 in *Sd*/+ embryos. No new notochord is formed caudally from these timepoints forward. The notochord that has developed degenerates completely in both *Sd/Sd* and *Sd/+* embryos by E14 [6], [7]. However, the floorplate that developed alongside the notochord prior to notochord degradation remains intact. Sonic hedgehog (*Shh*) expression is affected by the lack of notochord, though the floorplate that remains after notochord degeneration still expresses *Shh* normally [42]. The lack of notochord and floorplate in the most caudal portions of the embryo is a plausible explanation for some aspects of the characteristic *Sd* phenotype. However, the lack of notochord is the same in both homozygous and heterozygous embryos even though the phenotype is less severe in heterozygous animals, suggesting that simple lack of *Shh* in the caudal portion of the embryo is unlikely the sole cause of the *Sd* phenotype. It is more likely that a combination of lack of *Shh* from the absent notochord as well as misexpression of other genes due to the ectopic overexpression of *Ptf1a* play a significant role in the phenotypic etiology. Global gene expression analysis of various timepoints in developing *Sd* mutants will be helpful to characterize these changes.

Mouse models have been invaluable in the study of human development, and the Danforth's short tail mouse is a striking model of human caudal birth defects. The *Sd* mouse exhibits features seen in caudal regression syndrome (CRS), urorectal septum malformation sequence/persistent cloaca (URSMS), VACTERL association, and Currarino syndrome. Shared phenotypic characteristics include kidney dysgenesis (or agenesis), vertebral anomalies, and anorectal malformations. The overlap of phenotypic characteristics suggests that there are similar pathways or related pathways that are disrupted in these disorders. Interestingly, Currarino syndrome, characterized by the triad of hemisacrum, anorectal malformation and pre-sacral mass, is caused by mutations in the *HLXB9* gene (also known as *MNX1*), which is known to have a role in pancreas and motor neuron development [8], [9]. Recently, it has been reported that *Ptf1a* is a strong regulator driving *Mnx1* expression during pancreatic development [43]. Although we identified a significant increase in *Ptf1a* in mutant *Sd* embryos at E9.5, we did not observe a concurrent change in *Mnx1* expression in mutant embryos (data not shown). Further analysis of expression patterns may be needed at later timepoints to identify downstream changes due to *Ptf1a* misexpression. Importantly, mice lacking *Mnx1* do not mirror the caudal phenotype seen in human patients with Currarino syndrome [44]. Thus, the genetic pathways leading to caudal dysgenesis via misexpression of *Ptf1a* and/or *Mnx1* may differ between these species.

It is hypothesized that caudal malformation disorders result from failure of induction and proliferation of the caudal mesoderm [45]. In URSMS it is specifically postulated that the phenotype is caused by failure of the meosdermally derived urorectal septum to properly grow and divide the cloaca into the primary urogenital sinus (ventrally) and the rectum (dorsally) [46]. Studies of chimeric *Sd* mice in which *Sd* mutant cells carrying the LacZ gene were injected into wildtype blastocysts show specific exclusion of all *Sd* cells from the dorsal side of the urorectal septum (which overlaps the ventral portion of the hindgut), indicating a failure of this structure to grow in mutant animals [47]. Whether *Ptf1a* is overexpressed due to the ETn insertion in the developing mesoderm of the urorectal septum in *Sd* embryos is unknown and will need further investigation. Now that the genetic lesion has been identified, the study of downstream gene expression changes in *Sd* mice will undoubtedly lead to an improved understanding of human caudal development disorders.

## Materials and Methods

### Ethics statement

All experiments involving mice have been approved by The University of Michigan University Committee on Use and Care of Animals.

### Mice

The *Sd* mutation has been maintained at the Jackson Laboratories on a recombinant inbred strain (RSV/LeJ, stock# 000268) for over 127 generations, and we have established an *Sd* breeding colony at the University of Michigan. Data presented herein are from mice from the RSV/LeJ line, or from outcrossing the *Sd* mutation to CD-1 mice (Charles River Laboratories, Wilmington, MA). An outbred/mixed background was maintained by crossing inbred RSV/LeJ mice to CD-1 and harvesting embryos only from intercrossed F1 mice. The *Sd* phenotype is consistent between the inbred and outbred mice. Mice were housed in environmentally controlled conditions with 14 hour light/10 hour dark cycles with food and water provided *ad libitum*.

### Timed pregnancies

Matings for timed embryo isolation were set up using standard animal husbandry techniques. Noon on the day of vaginal plug observation was considered embryonic day (E) 0.5. DNA for embryo genotyping was isolated from yolk sacs via the HotSHOT extraction method.

### Genomic DNA isolation

Genomic DNA for next-generation sequencing and Southern analysis was isolated using phenol∶chloroform extraction and ethanol precipitation, and DNA was quantified on a NanoDrop spectrophotometer (ThermoFisher, Asheville, NC).

### Targeted capture, library creation, and next-generation sequencing

5 µg (at 50 ng/µl) of heterozygous *Sd* DNA was sheared into 300 bp fragments on a Covaris S-series sonicator (Covaris, Woburn, MA) and was subsequently used to create a total genomic DNA library for next-generation sequencing using standard Illumina protocols. Prior to sequencing, the DNA library was enriched for the *Sd* critical region (between chromosome 2 bases 18,883,257–20,481,194 on build 37 of the mouse genome (July 2007; mm9) as visualized on the UCSC genome browser (http://www.genome.ucsc.edu) using a 244 K Agilent SureSelect DNA oligo microarray designed utilizing Agilent eArray software (https://earray.chem.agilent.com/). Capture was performed by following the Agilent SureSelect Array protocol version 1. The captured DNA was tested for proper enrichment via quantitative PCR with locus specific primers and then subject to 36 bp paired-end sequencing on an Illumina Genome Analyzer IIx at the University of Michigan DNA Sequencing Core.

### Bioinformatic analysis of next-generation sequencing data

Next-generation sequence data were aligned to the corresponding region of chromosome 2 represented on the NCBI37/mm9 build of the mouse genome. Sequences were aligned using the Bowtie and MAQ software applications [16]. Overall assembly of the region was done using the Mosaik software suite and assembled discrepancies were mined using Consed software [48]. Sequences were also aligned using the Bowtie software application. Output files were produced in SAM and BAM format and converted to BED format for visualization on the UCSC Genome Browser. Histogram graphs to analyze coverage (copy number variation and deletions and duplications) were created.

The unmapped reads contain a combination of poor quality sequence and reads which couldn't be mapped to the existing reference sequence. Reasons for not mapping include repetitive and low complexity sequence as well as novel elements. Using the paired-end reads, and a combination of existing tools, and custom methods, we identified read pairs where there were at least 5 sequence reads where one end mapped uniquely to Chromosome 2 and the other end did not map but contained a repetitive element. We compared these repetitive sequences (LTR) with known locations of repeat sequences in the mouse genome to identify novel insertions and their locations.

### Genotyping

Multiplex PCR was used for genotyping with primers CNV577 (5′-TTTCCACGGCCATTCTTTAC-3′), CNV578 (5′-GCTCAACCAGAACAATACATTCAG-3′) and CNV580 (5′-GCCAATCAGGAGACTGAAGC-3′). PCR was performed in 20 µl reactions containing 1 µM of each primer, and 1× TaqProComplete (Denville Scientific Inc., Metuchen, NJ) in an Eppendorf Mastercycler (Eppendorf North America, Hauppauge, NY). Cycling conditions were 94°C for 5 minutes, followed by 30 cycles of 94°C for 30 seconds, 58°C for 30 seconds, and 72°C for 45 seconds, and a final extension of 72°C for 10 minutes. PCR products were resolved on a 2%TAE gel stained with ethidium bromide. The resulting wildtype amplimer is 512 bp and the *Sd* allele amplimer is 405 bp.

### Southern analysis

6 µg of genomic DNA from wildtype, *Sd/+*, and *Sd/Sd* was digested with 4 U/µg of either EcoRI or BamHI (New England Biolabs, Ipswich MA) supplemented with 2.5 mM spermidine overnight at 37°C. Digested DNA was electrophoresed on a 1% TAE gel and transferred to Hybond-N+ nylon membranes as previously described. Digoxigenin labeled probe was created using the Roche PCR DIG probe synthesis kit (Roche Applied Science, Indianapolis, IN) per manufacturer instructions with primers CNV551 (5′-AACCACAGGAAAGGTTGCAG-3′) and CNV552 (5′-TCTGGGTACCAGCTTCAGTG-3′) using mouse genomic DNA as a template. Southern analysis was performed using the Roche DIG Easy Hyb system per manufacturer instructions (Roche Applied Science, Indianapolis, IN).

### Long-range PCR and cloning

PCR primers flanking the *Sd* ETn insertion were designed using Primer3 software (http://frodo.wi.mit.edu/primer3/). The ETn was amplified using the Roche Expand Long Template PCR system (Roche Applied Science, Indianapolis, IN) per manufacturer instructions with primers CNV559 (5′-GAAGCTCTGCAGGCTGAAAGCAAAG-3′) and CNV560 (5′-GAATGAGGACTCTGCCCTTGAGTGG-3′). Amplified PCR product was cloned using the TOPO XL PCR cloning kit (Invitrogen/Life Technologies, Grand Island, NY). Sequencing of the cloned ETn was performed by primer walking (see GenBank Accession JX863104)

### Quantitative real-time PCR

Primers for qRT-PCR were identified via PrimerBank (http://pga.mgh.harvard.edu/primerbank/) for *Ptf1a*, *Msrb2*, *Otud1*, *Etl4*, *Pip4k2a*, and *Armc3*. Primer3 was used to design primer pairs for *4921504E06Rik* (CNV496 5′-TACCTAGCATGGTGCCTGAAGA-3′/CNV497 5′-GTCATTGCATACTGCCGGTAAA-3′), BQ085140 (CNV788 5′-GTGCTGGACCCAAACATAGC-3′/CNV789 5′-TGGGGAATCAACGAACTCTG-3′), and AK053418 (CNV792 5′-TAAGGGGATGGGAAGGTGTC/CNV793 5′-AGGTGCATCATCATGGCTTC-3′). cDNA template for qRT-PCR was synthesized using the First Strand cDNA synthesis kit from Invitrogen/Life Technologies (Grand Island, NY) from 1 µg RNA isolated from E9.5 *Sd/Sd*, *Sd/+*, and +/+ embryos (3 from each genotype) using the RNeasy Micro Kit (Qiagen, Valencia, CA). qRT-PCR reactions were performed in triplicate using 1× Applied Biosystems Power SYBR mix and run on an Applied Biosystems StepOne Plus PCR system (Applied Biosystems/Life Technologies, Grand Island, NY). Cycling conditions were 50°C for 2 minutes, 95°C for 10 minutes, followed by 40 cycles of 95°C for 15 seconds, 57°C for 30 seconds, 72°C for 30 seconds. The cycle threshold for each reaction was automatically calculated using StepOne Software v2.2. Fold change of the test genes was calculated for each genotype by normalization to β-actin using the Pfaffl method [49]. Statistical analysis was performed via Student's T-test with significance p<0.05.

### 5′ RACE

5′ RACE was performed using the FirstChoice RLM-RACE kit (Ambion/Life Technologies, Grand Island, NY) per manufacturer instructions with 1 µg template *Sd/Sd* RNA as input. RACE products were sequenced at the University of Michigan DNA Sequencing Core.

## Supporting Information

Figure S1 qRT-PCR of the captured DNA library that was used for next-generation sequencing. PCR analysis shows successful capture and enrichment of the *Sd* critical region. A) qRT-PCR results using primers mapping to the captured region post and pre-hybridization. B) qRT-PCR results using primers mapping outside the captured region pre- and post-hybridization.
(TIF)

Figure S2 *Sd* Transgene Constructs A) The 3.2 kb pCAGGS-*mPtf1a* transgene contains a 357 bp cytomegalovirus immediate-early enhancer (Enh), a 1266 bp intron, the 975 bp *Ptf1a* coding sequence (*Ptf1a-*CDS) and a 643 bp rabbit β-globin polyA (PolyA). B) Representation of the BAC-based genomic transgene clones. The colored bars represent the 40.4 kb transgene containing the *Sd* ETn on top and the associated 31.9 kb control transgene below. Exons of the *Ptf1a* gene are represented in blue, the conserved 5′ enhancer in red and 3′ control region in green [20]. The location of the *Sd* ETn is denoted by the triangle. The transgenes are indicated above a VISTA plot (http://pipeline.lbl.gov/cgi-bin/gateway2) showing conservation of the genomic region surrounding the transgenes between the 3′ ends of the *Msrb2* and 4921504E06Rik genes (Chr2:19,314,600–19,389,900; NCBI37/mm9 genome build) in various mammals. The location of the *Sd* ETn is indicated by an asterisk on the VISTA plot.
(TIF)

Table S1 Transgenic Mouse Analysis.
(DOCX)

Text S1 Supporting Materials and Methods used in creation of transgenic mice.
(DOCX)
