# Supplementary figures and images for: Next-Generation Sequencing Identifies the Danforth's Short Tail Mouse Mutation as a Retrotransposon Insertion Affecting Ptf1a Expression
Source: PLoS Genet. 2013 Feb 21;9(2):e1003205. doi: 10.1371/journal.pgen.1003205 (PMC3578742; doi:10.1371/journal.pgen.1003205)

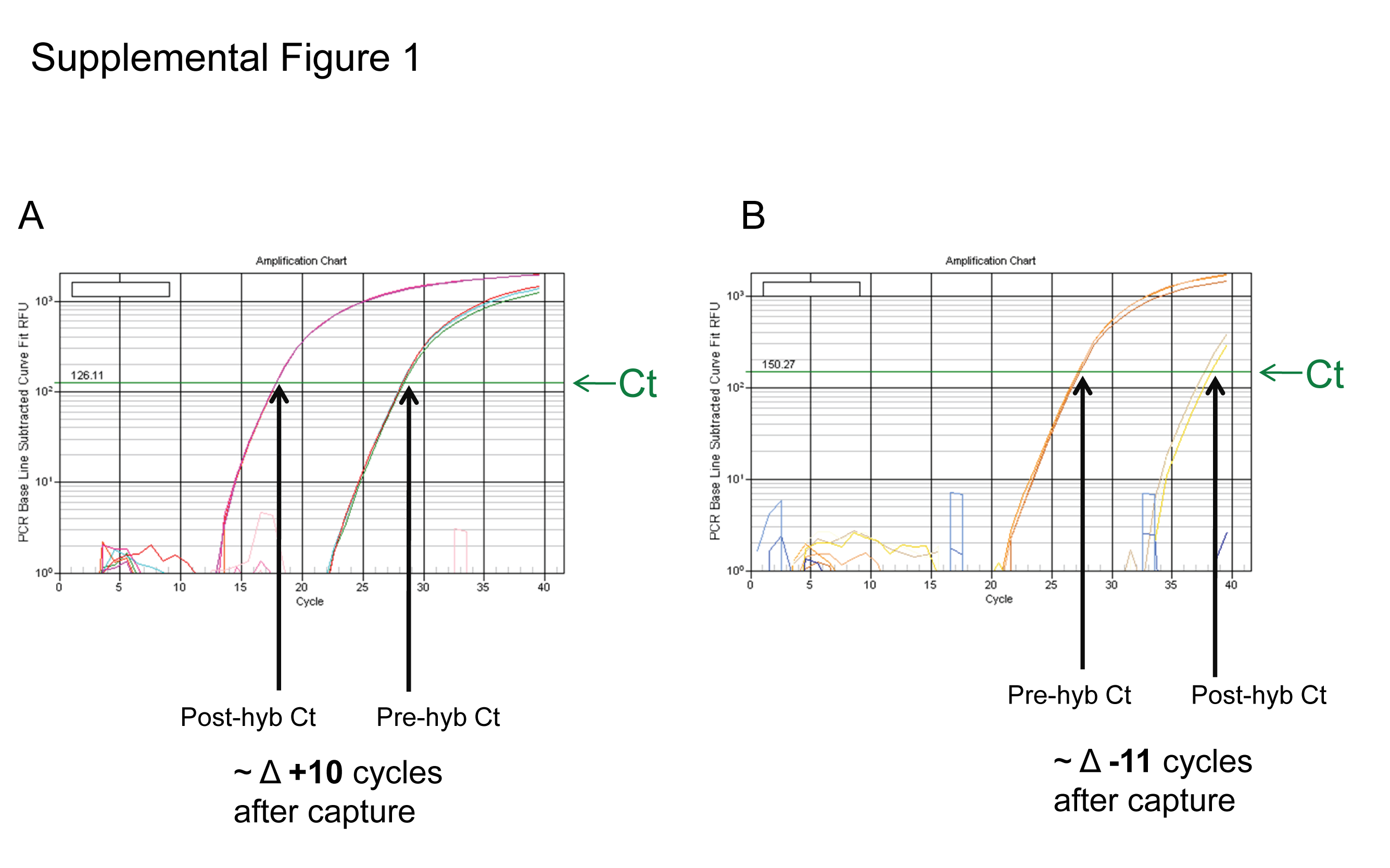

Supplement: Figure S1 — qRT-PCR of the captured DNA library that was used for next-generation sequencing. PCR analysis shows successful capture and enrichment of the Sd critical region. A) qRT-PCR results using primers mapping to the captured region post and pre-hybridization. B) qRT-PCR results using primers mapping outside the captured region pre- and post-hybridization. (TIF) [file pgen.1003205.s001.tif]

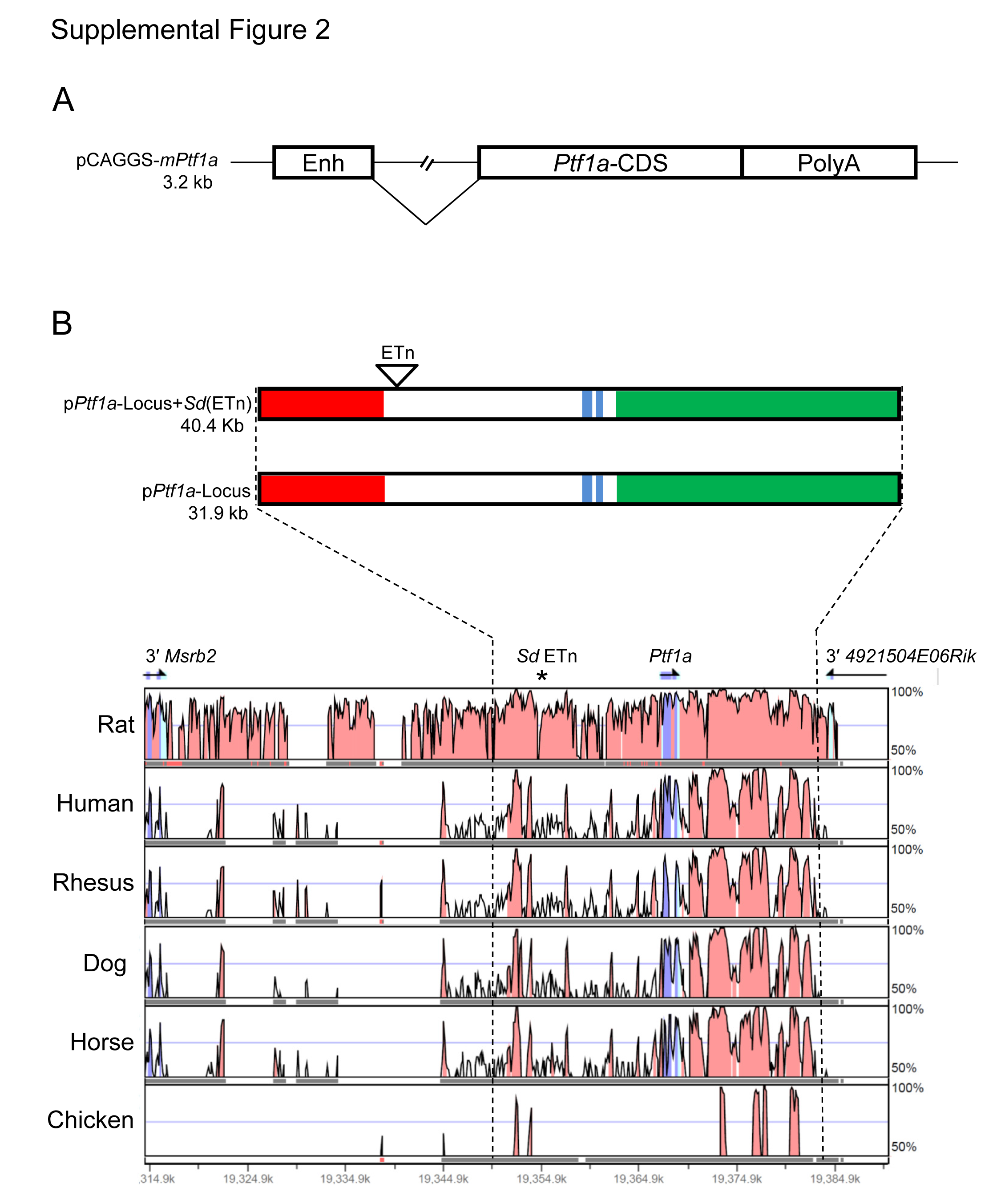

Supplement: Figure S2 — Sd Transgene Constructs A) The 3.2 kb pCAGGS-mPtf1a transgene contains a 357 bp cytomegalovirus immediate-early enhancer (Enh), a 1266 bp intron, the 975 bp Ptf1a coding sequence (Ptf1a-CDS) and a 643 bp rabbit β-globin polyA (PolyA). B) Representation of the BAC-based genomic transgene clones. The colored bars represent the 40.4 kb transgene containing the Sd ETn on top and the associated 31.9 kb control transgene below. Exons of the Ptf1a gene are represented in blue, the conserved 5′ enhancer in red and 3′ control region in green [20]. The location of the Sd ETn is denoted by the triangle. The transgenes are indicated above a VISTA plot (http://pipeline.lbl.gov/cgi-bin/gateway2) showing conservation of the genomic region surrounding the transgenes between the 3′ ends of the Msrb2 and 4921504E06Rik genes (Chr2:19,314,600–19,389,900; NCBI37/mm9 genome build) in various mammals. The location of the Sd ETn is indicated by an asterisk on the VISTA plot. (TIF) [file pgen.1003205.s002.tif]
