# Supplementary material for: Next-Generation Sequencing Identifies the Danforth's Short Tail Mouse Mutation as a Retrotransposon Insertion Affecting Ptf1a Expression
Source: PLoS Genet. 2013 Feb 21;9(2):e1003205. doi: 10.1371/journal.pgen.1003205 (PMC3578742; doi:10.1371/journal.pgen.1003205)
Supplement: Table S1 — Transgenic Mouse Analysis. (DOCX) [file pgen.1003205.s003.docx]

Table S1 Transgenic Mouse Analysis

| Transgene Name | Total # Embryos  Collected | # Embryos with transgene | # Embryos with phenotype |
| --- | --- | --- | --- |
| pCAGGS-m*Ptf1a* | 116 | 6^*^ | 0 |
| p*Ptf1a*-Locus | 32 | 16 | 0 |
| p*Ptf1a*-Locus+*Sd*(ETn) | 23 | 3^$^ | 0 |

^*^3 pCAGGS-*mPTF1a* transgenic embryos were living and not expressing the *Ptf1a* transgene, and

3 were growth arrested, dead, and unable to be tested for transgene expression.

^$^The number of embryos carrying the BAC-based p*Ptf1a-*Locus+*Sd*(Etn) transgene are significantly different than embryos carrying the associated p*Ptf1a-*Locus only control transgene (p=0.0087, Fisher’s exact test).
