# Supplementary material for: Next-Generation Sequencing Identifies the Danforth's Short Tail Mouse Mutation as a Retrotransposon Insertion Affecting Ptf1a Expression
Source: PLoS Genet. 2013 Feb 21;9(2):e1003205. doi: 10.1371/journal.pgen.1003205 (PMC3578742; doi:10.1371/journal.pgen.1003205)
Supplement: Text S1 — Supporting Materials and Methods used in creation of transgenic mice. (DOCX) [file pgen.1003205.s004.docx]

**Text S1**

**Supplementary Data Methods**

*pCAGGS-*mPtf1a *Transgenic Embryo Analysis*

The pCAGGS-*mPtf1a* transgene was created by first PCR amplifying the *Ptf1a* coding sequence from a sub-clone of EST I.M.A.G.E. clone 8861527 (GenBank accession no.BC138507). PCR products and the pCAGGS vector [[1](#_ENREF_1)] were doubly digested with BglII and EcoRI restriction enzymes (NEB, Ipswich, MA). Digested PCR product and pCAGGS vector were ligated with T4 ligase (NEB, Ipswich, MA), and sequence integrity of the resulting clone was confirmed via DNA sequence analysis. The pCAGGS-*mPtf1a* transgene was liberated from the vector sequence by digestion with SalI/StuI (NEB, Ipswich, MA). The transgene was purified and injected into fertilized (C57BL X SJL)F2 mouse eggs at the University of Michigan Transgenic Animal Model Core. After injection embryos were harvested at E16.5, analyzed for phenotype, and photographed. PCR genotyping for the transgene was performed on DNA isolated from yolk sac via the HotSHOT method with primers spanning intron 1 of the *Ptf1a* gene. Genotyping primers amplify a 181bp fragment from the transgene and a 512bp fragment from the endogenous *Ptf1a* genomic locus. RNA was isolated (when possible) from the forelimb and paw of pCAGGS-*mPtf1a* positive and negative littermates using the Qiagen RNeasy Kit (Qiagen, Valencia, CA). cDNA template for RT-PCR was synthesized using the First Strand cDNA synthesis kit from Invitrogen/Life Technologies (Grand Island, NY) from 1µg RNA. cDNA was diluted 1:20 and PCR was performed using primers specific to *Ptf1a.* All PCR primer sequences and PCR conditions for cloning, genotyping, and expression analysis of the pCAGGS-*mPtf1a* transgene are available from the authors upon request.

*Genomic* Ptf1a *+ Sd ETn Transgenic Embryo Analysis*

Two genomic *Ptf1a* genomic transgene constructs were created by bacterial artificial chromosome (BAC) recombineering as previously described [[2](#_ENREF_2),[3](#_ENREF_3)]. For the pPtf1a-locus plasmid a 31,932 bp genomic fragment spanning mouse chr2:19,351,125 to 19,383,056 (NCBI37/mm9) was sub-cloned from BAC RP24-347M17 into the pBR322 vector using gap repair as described [[2](#_ENREF_2)]. The *Sd* ETn was inserted into the p*Ptf1a*-Locus plasmid using recombineering in SW102 cells [[3](#_ENREF_3)] to create the 40,460 bp p*Ptf1a*-Locus+Sd(ETn) transgene. The integrity of both transgenes was thoroughly analyzed using a panel of restriction enzymes. Both transgenes were linearized with SgrA1 (NEB, Ipswich, MA) prior to injection into C57BL/SJL mouse eggs at the University of Michigan Transgenic Animal Model Core. After injection embryos were harvested between E14.5 and E15.5, analyzed for phenotype, and photographed. PCR genotyping for the transgene was performed using two primer pairs spanning the 5’ and 3’ ends of the transgenes to ensure complete integration. Fisher’s exact test (http://www.langsrud.com/fisher.htm) was performed to determine statistical significance between the number of embryos carrying the ETn containing transgene versus control. All PCR primer sequences used in cloning/recombineering and genotyping are available from the authors upon request.

**Supplementary References**

1. Niwa H, Yamamura K, Miyazaki J (1991) Efficient selection for high-expression transfectants with a novel eukaryotic vector. Gene 108: 193-199.

2. Liu P, Jenkins NA, Copeland NG (2003) A highly efficient recombineering-based method for generating conditional knockout mutations. Genome Res 13: 476-484.

3. Warming S, Costantino N, Court DL, Jenkins NA, Copeland NG (2005) Simple and highly efficient BAC recombineering using galK selection. Nucleic Acids Res 33: e36.
